# Sodium-Glucose Cotransporter 2 Inhibitors: Possible Anti-Atherosclerotic Effects Beyond Glucose Lowering

**DOI:** 10.14740/jocmr2385w

**Published:** 2015-12-03

**Authors:** Hidekatsu Yanai, Hisayuki Katsuyama, Hidetaka Hamasaki, Hiroki Adachi, Sumie Moriyama, Reo Yoshikawa, Akahito Sako

**Affiliations:** a Department of Internal Medicine, National Center for Global Health and Medicine Kohnodai Hospital, Chiba, Japan

**Keywords:** Atherosclerosis, Blood pressure, Body weight, Glucagon, Sodium-glucose cotransporter 2 inhibitor

## Abstract

The new drug for type 2 diabetes, the sodium-glucose cotransporter 2 (SGLT-2) inhibitor, is reversible inhibitor of SGLT-2, leading to reduction of renal glucose reabsorption and decrease of plasma glucose, in an insulin-independent manner. In addition to glucose control, the management of coronary risk factors is very important for patients with diabetes. Here we reviewed published articles about the possible anti-atherosclerotic effects beyond glucose lowering of the SGLT-2 inhibitors. We searched by using Pubmed, and found 770 published articles about SGLT-2 inhibitors. Among 10 kinds of SGLT-2 inhibitors, the number of published articles about dapagliflozin was the greatest among SGLT-2 inhibitors. Since SGLT-2 inhibitors have similar chemical structures, we concentrated on the published articles about dapagliflozin. SGLT-2 inhibitors are proved to be significantly associated with weight loss and reduction of blood pressure by a relatively large number of studies. The studies investigating effects of dapagliflozin on visceral fat, insulin sensitivity, serum lipids, inflammation and adipocytokines are very limited. An influence of increase in glucagon secretion by SGLT-2 inhibitors on metabolic risk factors remains unknown.

## Introduction

Sodium-glucose cotransporter 2 (SGLT-2) mediates approximately 90% of active renal glucose reabsorption in the proximal tubule of the kidney [1]. Recently, the new drug for type 2 diabetes, the SGLT-2 inhibitor was developed. The SGLT-2 inhibitor is reversible inhibitor of SGLT-2, leading to reduction of renal glucose reabsorption and decrease of plasma glucose, in an insulin-independent manner [2]. Diabetes is a strong independent risk factor for cardiovascular diseases (CVDs) [3]. Compared with subjects without diabetes, the relative risk for CVD is 2 - 3 times greater in men with diabetes and 3 - 4 times greater in women with diabetes [4-10]. In addition to glucose control, the management of coronary risk factors is very important for patients with diabetes. Here we reviewed published articles about the possible anti-atherosclerotic effects beyond glucose lowering of the SGLT-2 inhibitors.

## The Search Strategy for Published Articles About the Anti-Atherosclerotic Effects Beyond Glucose Lowering of the SGLT-2 Inhibitors

We searched by using Pubmed (Table 1), and found 770 published articles about SGLT-2 inhibitors. Ten kinds of SGLT-2 inhibitors were detected, and we searched the published articles about each SGLT-2 inhibitor. The number of published articles about dapagliflozin was the greatest among SGLT-2 inhibitors. Since SGLT-2 inhibitors have similar chemical structures, we concentrated on the published articles about dapagliflozin.

**Table 1:** The Reported Sodium Glucose Cotransporter 2 Inhibitors

The search strategies by Pubmed	Published articles (n)
Sodium glucose cotransporter 2 inhibitor OR sodium glucose cotransporter 2 inhibitors OR SGLT2 inhibitor OR SGLT2 inhibitors OR SGLT-2 inhibitor OR SGLT-2 inhibitors	770
Each sodium glucose cotransporter 2 inhibitors	
Dapagliflozin	300
Canagliflozin	234
Empagliflozin	161
Ipragliflozin	42
Luseogliflozin	23
Tofogliflozin	23
Remogliflozin	15
Sergliflozin	15
Ertugliflozin	4
Sotagliflozin	3

## Glucose, Body Weight and Blood Pressure Lowering Effects of Dapagliflozin

Dapagliflozin also reduces renal glucose reabsorption and decrease of plasma glucose, in an insulin-independent manner [2], which induces reduction of body weight and blood pressure. Reduction of body weight and blood pressure by SGLT-2 inhibitors is also induced by osmotic diuretics [11]. There were 106 published articles about “dapagliflozin and body weight” and 78 articles about “dapagliflozin and blood pressure”.

Matthaei et al studied effects of dapagliflozin 10 mg/day or placebo for 52 weeks on metabolic parameters in patients with type 2 diabetes using sulphonylurea and metformin [12], HbA1c and fasting plasma glucose levels showed greater improvement from baseline with dapagliflozin (-0.8% and -1.5 mmol/L) than with placebo. Dapagliflozin was associated with greater reductions in body weight and systolic blood pressure (-2.9 kg and -1.0 mm Hg) compared with placebo. Dapagliflozin was administered as monotherapy (n = 249) or combination therapy (n = 479) with existing antihyperglycemic agents to Japanese patients with diabetes for 52 weeks [13]. In patients receiving dapagliflozin as monotherapy or combination therapy, reductions from baseline were observed in HbA1c (-0.7% in both groups), weight (-2.6 and -2.1 kg, respectively), and systolic blood pressure (-5.2 and -3.9 mm Hg). Dapagliflozin reduced body weight and blood pressure by both monotherapy and add-on therapy.

In a meta-analysis including all trials with a duration of at least 12 weeks, comparing an SGLT-2 inhibitor with a non-SGLT-2 inhibitor agent in type 2 diabetes, SGLT-2 inhibitors are effective in the treatment of type 2 diabetes, providing additional benefits, such as weight loss, reduction of blood pressure [14].

## Anti-Atherosclerotic Effects Beyond Glucose Lowering of Dapagliflozin

**Figure 1:**
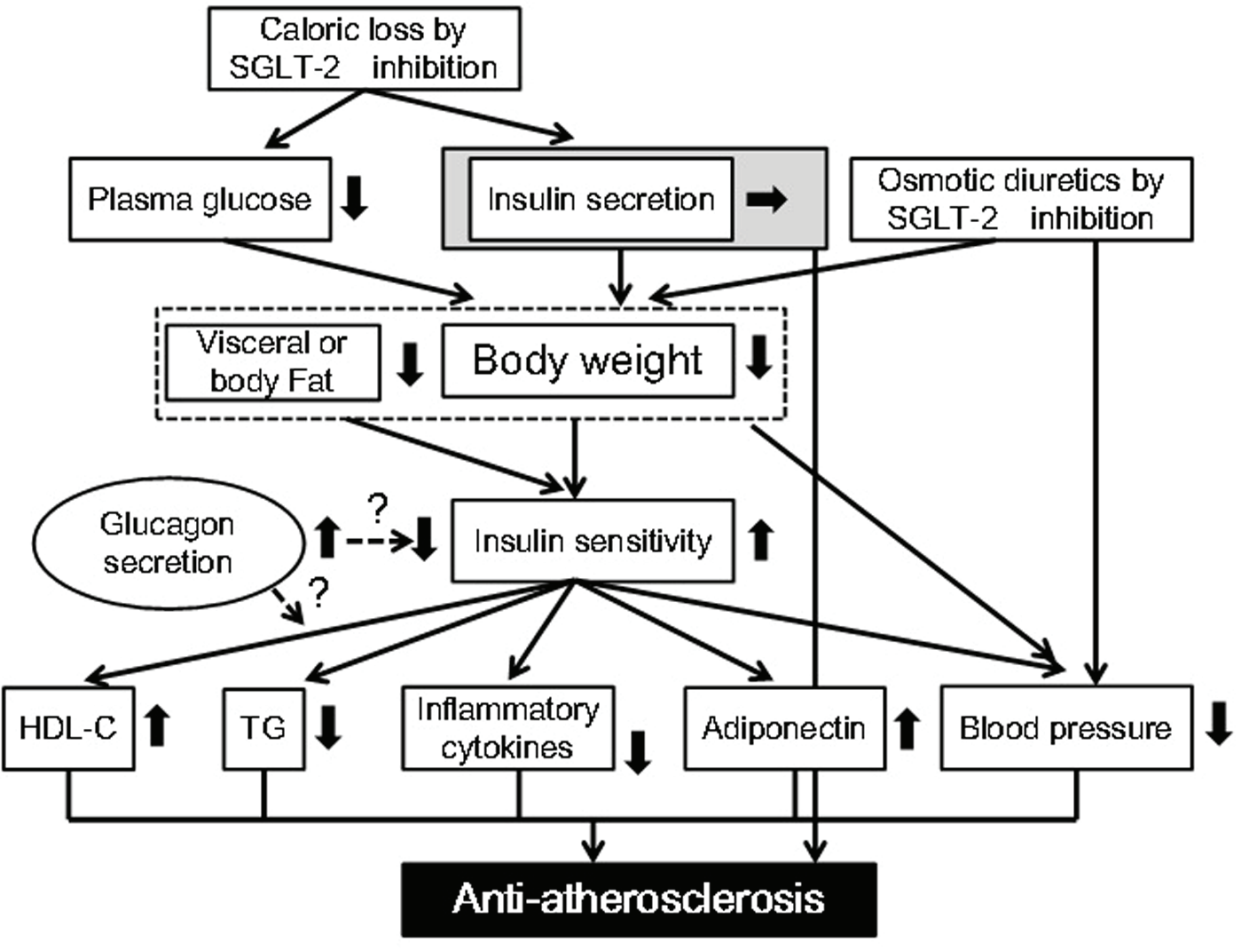
Possible anti-atherosclerotic effects beyond glucose lowering of sodium glucose cotransporter 2 inhibitors. HDL-C: high-density lipoprotein-cholesterol; SGLT-2: sodium glucose cotransporter 2; TG: triglyceride.

Improvement in glucose control, body weight and blood pressure by dapagliflozin was almost confirmed by a relatively large number of studies. We hypothesized the underlying mechanisms for possible anti-atherosclerotic effects beyond glucose lowering of SGLT-2 inhibitors (Fig. 1). We searched the published articles about the effects of dapagliflozin on metabolic risk factors by using Pubmed (Table 2). In this search, we excluded “Original Articles using animals or cells”, “Narrative Reviews” and “Expert Opinions”, and we considered “Original Articles”, Systematic Reviews” and “Meta-analysis” as important information.

**Table 2:** The Search Strategy to Find the Anti-Atherosclerotic Effects of Dapagliflozin

The search strategy by using Pubmed	Published articles (n)
Dapagliflozin and body weight	106
Dapagliflozin and blood pressure	78
Dapagliflozin and body fat OR dapagliflozin and visceral fat OR dapagliflozin and waist circumference OR dapagliflozin and abdominal circumference	4
Dapagliflozin and insulin resistance OR dapagliflozin and insulin sensitivity	38
Dapagliflozin and low density lipoprotein OR dapagliflozin and LDL	5
Dapagliflozin and high density lipoprotein OR dapagliflozin and HDL	6
Dapagliflozin and triglyceride	4
Dapagliflozin and adiponectin	0
Dapagliflozin and CRP OR dapagliflozin and C-reactive protein	1
Dapagliflozin and tumor necrosis factor alpha OR dapagliflozin and TNF-α	0
Dapagliflozin and interleukin-6 OR dapagliflozin and IL-6	0
Dapagliflozin and cytokine OR dapagliflozin and cytokines	2

### The effect of dapagliflozin on visceral or body fat

Patients (N = 182) were randomly assigned to receive dapagliflozin 10 mg/day or placebo added to open-label metformin [15]. Over 102 weeks, dapagliflozin-treated patients showed reductions in waist circumference by -5.0 cm and fat mass by -2.8 kg. In another study, 182 patients with diabetes were allocated to receive dapagliflozin 10 mg/day or placebo added to open-label metformin for 24 weeks [16]. Placebo-corrected changes with dapagliflozin were as follows: waist circumference, -1.52 cm (95% CI = -2.74 to -0.31; P = 0.0143); fat mass, -1.48 kg (95% CI = -2.22 to -0.74; P = 0.0001); visceral adipose tissue, -258.4 cm^3^ (95% CI = -448.1 to -68.6; nominal P = 0.0084).

### The effect of dapagliflozin on insulin sensitivity

We selected “Original Articles” which evaluated insulin sensitivity using the euglycemic hyperinsulinemic clamp. Twenty-four subjects with diabetes received dapagliflozin (n = 16) or placebo (n = 8) for 2 weeks, and the euglycemic hyperinsulinemic clamp was performed before and after treatment [17]. Dapagliflozin significantly improved whole-body insulin sensitivity. In another study, 18 diabetic men were randomized to receive either dapagliflozin (n = 12) or placebo (n = 6) for 2 weeks [18]. Improvement in muscle insulin sensitivity by dapagliflozin was observed by using the euglycemic hyperinsulinemic clamp. Forty-four subjects were randomized to receive dapagliflozin 5 mg or matching placebo once daily for 12 weeks [19]. Insulin sensitivity was assessed by measuring the glucose disappearance rate during the last 40 min of a 5-h euglycemic hyperinsulinemic clamp. Dapagliflozin treatment improved insulin sensitivity.

### The effect of dapagliflozin on serum lipids

In the study by Matthaei et al [12], adjusted % changes of fasting LDL-cholesterol from baseline to week 52 (95% CI) in the placebo group and the dapagliflozin group were 0.9 (-6.7, 9.1) and 4.8 (-1.5, 11.5), respectively. Adjusted % changes in fasting HDL-cholesterol were 0.6 (-3.6, 4.9) and 6.9 (3.3, 10.6) in the placebo group and the dapagliflozin group, respectively. Adjusted % changes in fasting LDL/HDL ratio were 0.9 (-7.8, 10.5) and -2.5 (-9.3, 4.7). Adjusted % changes in fasting triglyceride were 2.9 (-8.1, 15.2) and -8.0 (-16.0, 0.7). This study suggested that dapagliflozin is beneficially associated with serum lipids. Matthaei et al also evaluated the efficacy and safety of a 24-week dapagliflozin treatment in patients with type 2 diabetes inadequately controlled with metformin and sulfonylurea [20]. Patients receiving dapagliflozin showed placebo-subtracted increases in total, LDL, and HDL-cholesterol (11.4 mg/dL, P = 0.0091; 11.4 mg/dL, P = 0.0030; 2.2 mg/dL, P = 0.0172, respectively) with no change in LDL/HDL ratio (0.1; P = 0.2008) or triglycerides (-16.5 mg/dL; P = 0.1755). This study did not show evident beneficial effects of dapagliflozin for serum lipids, except for an increase of HDL-cholesterol. In a meta-analysis of randomized clinical trials, SGLT-2 inhibitors determined a modest but statistically significant increase in HDL-cholesterol, with no effect on triglyceride and on total and LDL-cholesterol [14].

### The effect of dapagliflozin on inflammation and adipocytokines

In the review which critically assessed the results of up-to-date clinical trials with dapagliflozin [21], it was described that high sensitivity C-reactive protein levels were decreased in dapagliflozin-treated patients. We could not find published articles about effects of dapagliflozin on adipocytokines including interleukin-6, tumor necrosis factor alpha and adiponectin.

### The effect of dapagliflozin on glucagon secretion

In the study by Merovci et al [18], insulin-mediated tissue glucose disposal increased by approximately 18% after 2-week dapagliflozin treatment, while placebo-treated subjects had no change in insulin sensitivity. Following dapagliflozin treatment, an increase in fasting plasma glucagon concentration was observed. Glucagon is associated with insulin resistance [22]. An influence of increase of glucagon secretion by SGLT-2 inhibitors on insulin resistance or metabolic risk factors should be carefully observed.

## Conclusion

SGLT-2 inhibitors seem to be associated with weight loss and reduction of blood pressure by a relatively large number of studies. However, the studies that investigated effects of dapagliflozin on visceral fat, insulin sensitivity, serum lipids, inflammation and adipocytokines are very limited. Furthermore, an influence of increase in glucagon secretion by SGLT-2 inhibitors on metabolic risk factors remains unknown. The glucose lowering effect in an insulin-independent manner of SGLT-2 inhibitors prevents hyperinsulinemia, which may also contribute to anti-atherogenesis. Very recently, Zinman et al showed that the addition of another SGLT-2 inhibitor, empagliflozin, reduced rates of death from cardiovascular causes (38% relative risk reduction), hospitalization for heart failure (35%), and death from any cause (32%) [23], suggesting a possible anti-atherosclerotic effect of SGLT-2 inhibitors. However, further studies should be performed to elucidate anti-atherosclerotic effect of SGLT-2 inhibitors.
